# Sea Bindweed Prevents Mycotoxin Intoxication Through Antioxidant, Anti-Inflammatory and Cytoprotective Activities

**DOI:** 10.3390/toxins18030127

**Published:** 2026-03-02

**Authors:** Nolwenn Hymery, Halima Boussaden, Stéphane Cérantola, Xavier Dauvergne, Christian Magné

**Affiliations:** 1 EA 3882 Laboratoire Universitaire de Biologie et Ecologie Microbienne, Université de Bretagne Occidentale, CS 93837, F-29238 Brest, France; nolwenn.hymery@univ-brest.fr (N.H.);; 2 Laboratoire Géoarchitecture_Territoires, Urbanisation, Biodiversité, Environnement, Université de Bretagne Occidentale, CS 93837, F-29238 Brest, France; xavier.dauvergne@univ-brest.fr; 3 Service Général des Plate-Formes Technologiques, Plateforme Résonance Magnétique Nucléaire–Résonnance Paramagnétique Electronique, Université de Bretagne Occidentale, CS 93837, F-29238 Brest, France; stephane.cerantola@univ-brest.fr

**Keywords:** anti-inflammatory activity, antioxidant activity, cytotoxicity, halophytes, in vitro, mycotoxins, sea bindweed

## Abstract

Mycotoxins are the most frequently occurring natural contaminant in food and feed products. Through the deployment of diverse agricultural strategies or biological, chemical, or physical treatments of crop products, mycotoxin contamination remains a persistent issue for the agricultural sector and food/feed industry. We previously suggested that halophytes, thanks to their high antioxidant activity, could protect animal cell lines from mycotoxin contamination. Here, a hydroalcoholic extract of *Calystegia soldanella* L. leaves was evaluated for in vitro total antioxidant capacity (TAC) and 2,2-diphenyl-1-picrylhydrazyl (DPPH)-quenching bioassays, as well as anti-inflammatory (ELISA measurement of IL-8 secretion), ROS-inhibiting production (CellROX Green assay), and calcium influx restoration (fluorescent probe Fura2-QBT assay) activities in two animal cells upon mycotoxin intoxication. *C. soldanella* extract displayed high antioxidant activities (DPPH IC_50_ < 80 μg·mL^−1^ and TAC of 90 mg AAE·g^−1^ DW. Moreover, it exhibited a significant protective action on renal (MDBK) and intestinal (IPEC-J2) cells against zearalenone (ZEA) or T2-toxin contamination, restoring about 75% of cell viability (MTS bioassay) at 1 μg·mL^−1^. This effect was accompanied by strong anti-inflammatory, ROS-inhibition, and membrane integrity restoration activities. A bio-guided study revealed that the fraction of *C. soldanella* extract eluted from C18-bound silica with 60% methanol was the most active one. Upon HPLC and 1D- and 2D-NMR analyses, major compounds identified in this fraction were flavonol-type flavonoids, including quercetin-3-*O*-glucose (X1), quercetin-3-*O*-rutinoside (X2), and quercetin-3-*O*-glucose-6″-acetate (X3). Enriched sub-fractions containing these compounds largely contributed to the cytoprotective effects of *C. soldanella*, supporting its potential use as a food/feed ingredient.

## 1. Introduction

The feed supply chain is a critical component of all livestock production systems. According to FEFAC [1], approximately 640 million tons of feed materials and forages are consumed annually by livestock in the European Union. However, despite their nutritional value and economic importance, cereal grains, as well as vegetables and fruits, are particularly vulnerable to contamination by mycotoxins. These compounds are secondary metabolites produced by various fungal species, mainly of *Fusarium*, *Aspergillus*, and *Penicillium* genera. Mycotoxins are the most frequently occurring natural contaminant in food and feed [2], and they are frequently detected in crops under field conditions and post-harvest storage. Globally, it is estimated that about 25% of agricultural products and up to 50% of cereal grains or their derivatives used in animal feed are contaminated with at least one mycotoxin [3,4,5].

In compound feed, the most frequently detected mycotoxins under warm climate conditions are: fumonisins (91%), deoxynivalenol (DON, 82%), zearalenone (ZEA) (35%), aflatoxins (18%), T2 toxin (4%), and ochratoxin A (3%) [6,7]. While ZEA and DON are produced both in the field and during post-harvest storage (grains, silage), T2 toxin is only produced during seed storage. All three are toxic to animals, especially to monogastric species, which lack a rumen and its mycotoxin-degrading microbiota [8]. Zearalenone mimics estrogen and disrupts reproductive functions, inducing infertility or abortions [9,10]. T2 toxin inhibits protein and DNA synthesis, leading to weight loss, diarrhea, immune suppression, skin necrosis, and hemorrhage [11,12]. DON, also known as vomitoxin, impairs feed intake, weight gain, and vaccine efficacy, and can induce vomiting and feed refusal [8,13,14].

On the other hand, the deployment of field-level strategies such as crop rotation, varietal selection, irrigation, tillage, and fungicide applications can help reduce fungal growth and mycotoxin levels, and fungal presence and toxin contamination in cereals, vegetables, and fruits are still commonly observed. Alternatively, adsorbent materials such as clay-based products, activated charcoal, or biopolymers derived from algae have been used with varying degrees of success to reduce the bioavailability of mycotoxins during storage, while proving effective in vitro [15]. Some other chemical methods—notably ammoniation treatment or gamma irradiation—have been investigated, but these approaches are insufficiently effective to remove fusariotoxins or are impractical for routine application on farms [16,17]. More recently, efforts have focused on the use of probiotic microorganisms and their by-products as potential detoxifying agents [18], but their effectiveness remains limited, especially against deoxynivalenol (DON) [19]. As a result, mycotoxins are still a major challenge for livestock and poultry production systems, and there is a critical need for efficient, natural, and reliable strategies to safeguard livestock from the harmful impacts of these toxins.

Interest in plant-derived bioactives has increased with the current trend toward natural health solutions. Thus, phytoproducts are used extensively in medicine, nutraceuticals, and the food industry. Over the past 20 years, antioxidant-rich plants in particular have garnered a growing interest, and food supplements with exogenous antioxidants are considered promising to lessen the negative consequences of inflammation and oxidative stress, the two main issues caused by mycotoxin exposure [20]. Therefore, anti-inflammatory and antioxidant-rich plants might be efficient in mitigating mycotoxin contamination issues. In that context, the potent protective effect of salt-tolerant plants, also called halophytes, was investigated since they are rich in health-promoting substances with anti-inflammatory and antioxidant properties [21]. From a preliminary screening of about twenty halophytic plants, three species, including the herbaceous Convolvulaceae *Calystegia* (ex. *Convolvulus*) *soldanella*, were shown to prevent animal cells from intoxication with fungal toxins [22]. Although Convolvulaceae’s therapeutic benefits are not well known, some of its members have long been utilized in coastal folk medicines as a mild laxative agent, as it has diuretic, purgative, anti-obesity, and soothing properties [23,24]. More recently, *Calystegia soldanella* was demonstrated to have analgesic and antiangiogenesis properties and may find use in the treatment of nervous system and skin hazards [25,26]. Here, the effect of the halophyte *Calystegia soldanella* against mycotoxin contamination of animal cells is described, with the aim of highlighting the likely mechanism of plant effect and identifying the potent bioactive compounds responsible for this action.

## 2. Results

### 2.1. Antioxidant Activities of C. soldanella Extract and Fractions

*C. soldanella* leaf extract showed an appreciable antioxidant capacity with a TAC value of 89.1 mg AAE·g^−1^ DW (Table 1). Following the fractionation of the raw extract, this capacity was concentrated in four intermediate fractions, namely MeOH_20_ to MeOH_80_. The same trend was obtained with the 2,2-diphenyl-1-picrylhydrazyl (DPPH) assay, with an IC_50_ value of 77.5 ± 6.0 μg·mL^−1^ for the raw extract, where MeOH_20_ to MeOH_80_ fractions were the most active ones. Particularly, the MeOH_60_ fraction showed a strong antiradical action, with a 6-times lower IC_50_ (13 μg·mL^−1^) than that of the raw extract.

### 2.2. Cytotoxicity of Mycotoxin and Cell Protection by C. soldanella Extract

#### 2.2.1. Effects of Mycotoxins and Plant Extract on the Cell Viability

*C. soldanella* raw extract showed no cytotoxic effect on either renal (MDBK) or intestinal (IPEC-J2) cells at concentrations up to 1 µg·mL^−1^.

MDBK cells exposed to deoxynivalenol (DON), T2, or zearalenone (ZEA) mycotoxins underwent a marked loss of viability (40, 49, and 38%, respectively) (Table 2). However, preincubation with plant extract (1 µg·mL^−1^) significantly mitigated cytotoxicity caused by T2 and ZEA toxins (by 46 and 35%, respectively). Therefore, about three-quarters of MDBK cells preincubated with *C. soldanella* extract, then exposed to T2 or ZEA, were viable vs. only 51 and 61% without plant extract. Concerning DON cytotoxicity, no statistical differences were observed for cells cultured with or without plan extract (61% of viability).

Similar observations were made with the intestinal porcine cell line. Thus, exposition of IPEC-J2 cells to DON, T2, or ZEA toxin drastically decreased viability (to 52%, 62%, and 60%, respectively) (Table 2). Preincubation of the cells with sea bindweed extract significantly attenuated the viability loss caused by T2 or ZEA treatment, leading to 50 and 32% recovery, respectively. Here again, plant extract had no effect against DON intoxication.

#### 2.2.2. Effects of Mycotoxins and Plant Extract on the Inflammation Process

Preincubation of bovine or porcine cells with *C. soldanella* extract alone at concentrations up to 1 µg·mL^−1^ did not induce interleukine 8 production.

Both MDBK and IPEC-J2 cells exposed to DON, ZEA, or T2 toxins exhibited inflammation, as shown by the production of IL-8 (Table 3). T2 appeared as the most inflammatory toxin, inducing a 7- and 5-fold Il-8 secretion in MDBK and IPEC-J2 cells, compared to untreated ones. However, a preincubation with plant extract drastically alleviated this effect, as shown by similar IL-8 levels to those in untreated cells.

#### 2.2.3. Effects of Mycotoxins and Plant Extract on the Production of ROS

**Figure 1:**
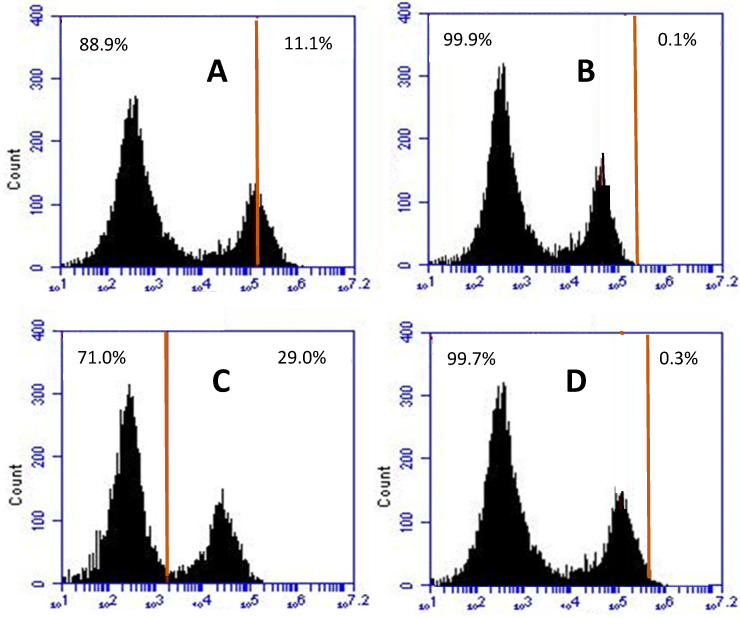
Effects of mycotoxins on ROS production in IPEC-J2 cells. Cells were treated with DON (**A**) or T2 toxins (**C**) alone, or pre-incubated for 24 h with *C. soldanella* extract before mycotoxin exposure (**B**,**D**). The black areas represent the fluorescence signal emitted by the cells (see Section 5.5.4), The fraction of ROS produced upon each treatment corresponds to the part of the fluorescent signal to the right of the red line, and data at the top right of each square indicates this fraction (0% corresponds to the absence of ROS formation by control untreated cells).

No oxidative stress was observed in intestinal cells in the presence of crude *C. soldanella* extract alone. Exposition of IPEC-J2 cells to DON and T2 toxins induced ROS formation by 11% and 29%, respectively, compared to the control (Figure 1A,C). Conversely, ZEA did not significantly cause ROS formation. A preincubation of cells with plant extract before DON or T2 exposure maintained the control level of ROS formation (Figure 1B,D).

#### 2.2.4. Effects of Mycotoxins and Plant Extract on Calcium Fluxes

A drop in calcium influx was observed in IPEC-J2 cells upon DON exposure, as shown by a marked decrease (35%) in the store-operated calcium channel activation (Table 4). Prior incubation with *C. soldanella* extract maintained the calcium influx at levels comparable to untreated cells. Cell exposure to T2 markedly blocked the SOC channel (−49%), but plant extract failed to maintain calcium influx. ZEA was the most SOC-inhibiting toxin (−66%), and plant extract partially prevented this effect.

#### 2.2.5. Effects of Mycotoxins and Plant Extract on the Cell Membrane Integrity

Trans-epithelial electrical resistance (TEER) was assessed in IPEC-J2 cells upon mycotoxin exposure (Table 5). In the presence of *C. soldanella* extract alone, TEER was not affected. However, exposure to DON, ZEA, and T2 toxins dramatically affects intestinal cell membrane integrity, decreasing TEER by 21, 25, and 34%, respectively. Preincubation with *C. soldanella* extract significantly mitigated this membrane impairment, with TEER being maintained close to that of untreated cells. Similar results were obtained for mitochondrial activity, evaluated with the MTS assay.

#### 2.2.6. Effects of Plant Fractions on Cell Viability upon Mycotoxin Exposure

**Figure 2:**
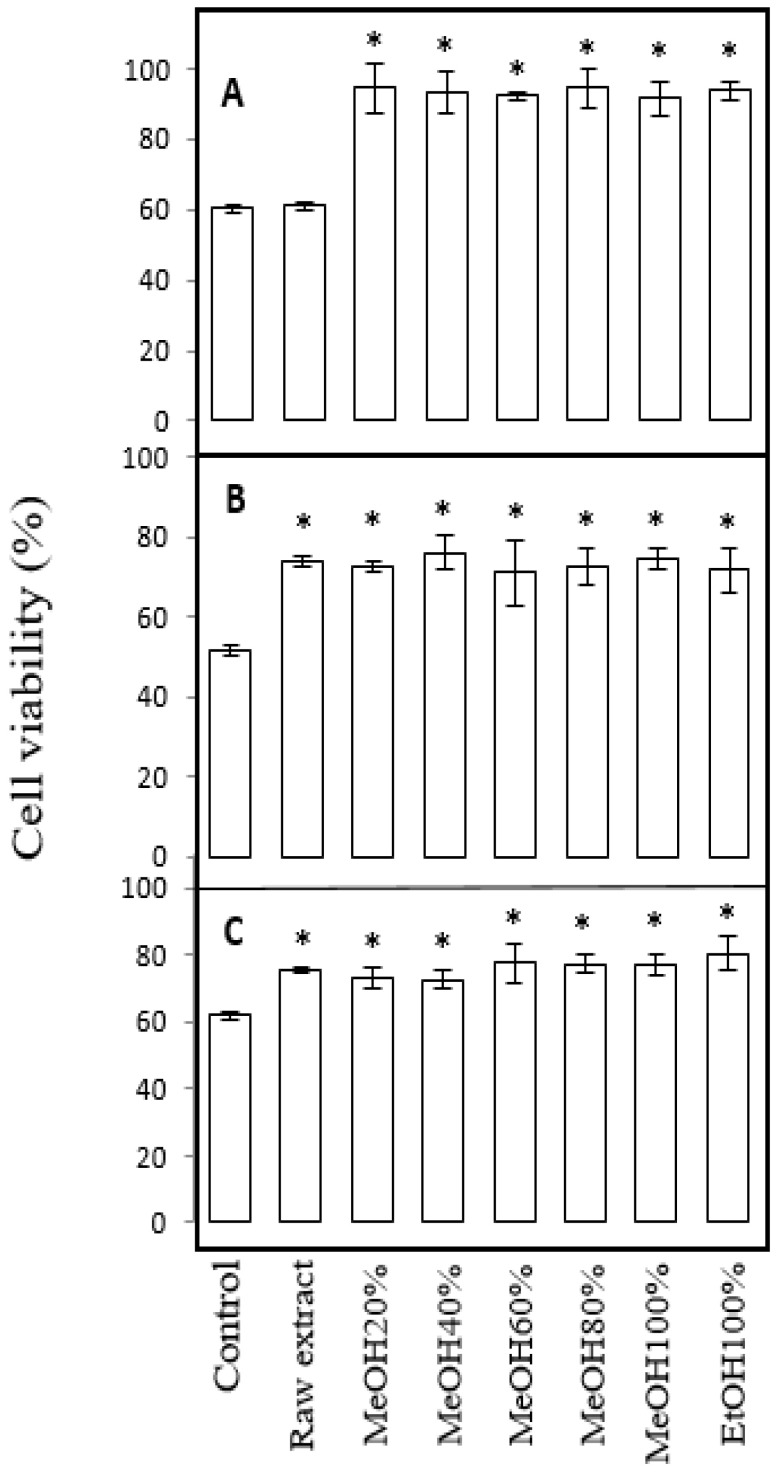
Effect of fractions of *C. soldanella* leaf extract on IPEC-J2 cell viability upon exposure to DON (**A**), T2 (**B**), or ZEA (**C**) mycotoxins. Mean ± SD of 3 replicates are represented; above bars, a asterisk indicates a significant difference with the mycotoxin control at *p* < 0.05.

Though *C. soldanella* raw extract had no effect on the viability of MDBK cells treated with DON, its fractions significantly attenuated the loss of viability (Figure 2A). Interestingly, no significant differences were found between the fractions, so that every fraction showed a similar level of protective activity. Upon T2 (Figure 2B) or ZEA (Figure 2C) exposure, cell viability was maintained only in part by plant fractions, with the same efficiency as the raw extract.

### 2.3. Elucidation of Major Constituents of Calystegia soldanella Extract

**Figure 3:**
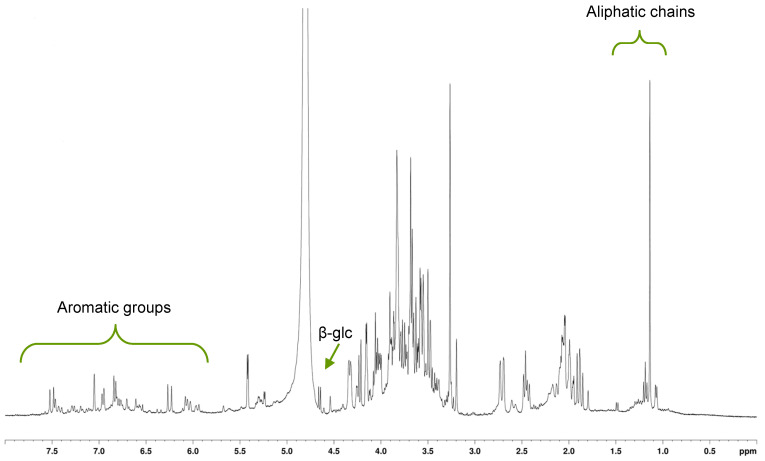
^1^H NMR spectrum of raw ethanol extract of *C. soldanella* leaves.

Characterization of *C. soldanella* extract and its fractions was performed by nuclear magnetic resonance (NMR) spectroscopy. Accordingly, the ^1^H-NMR spectrum of the raw extract showed the presence of carbohydrates, and several signals in the aromatic and aliphatic zones (5.7–7.7 ppm and 1–2 ppm, respectively) (Figure 3). 

**Figure 4:**
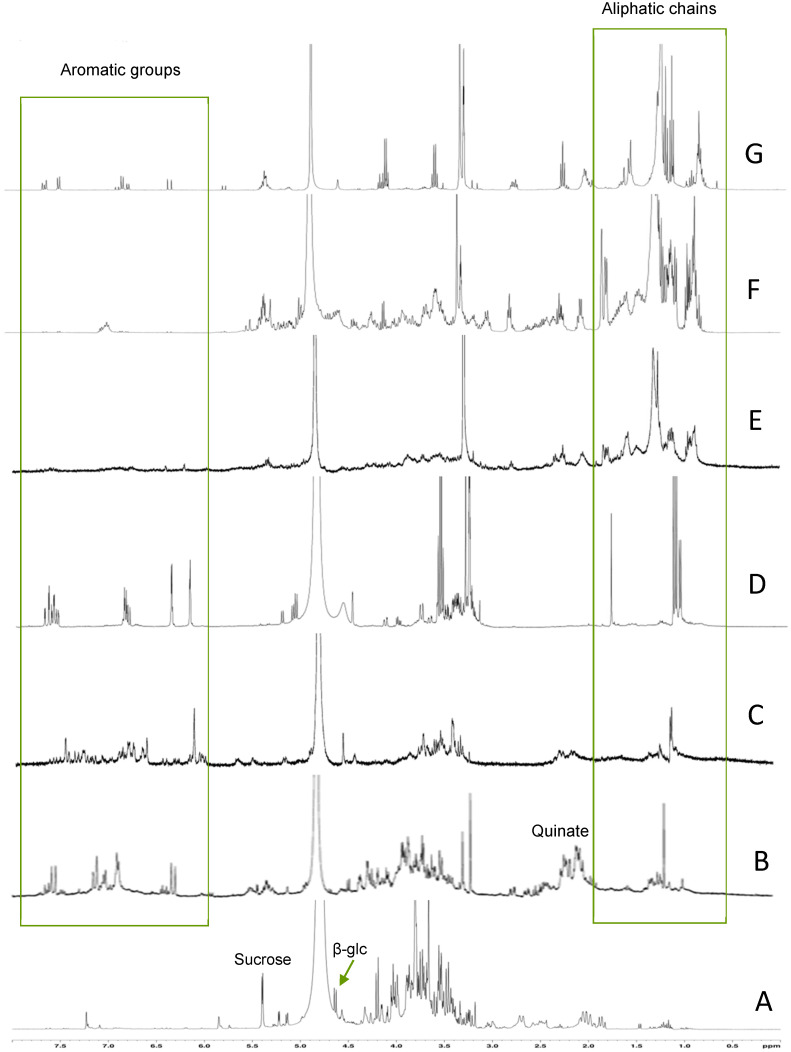
^1^H NMR spectrum of *C. soldanella* fractions eluted with water (**A**), 20% MeOH (**B**), 40% MeOH (**C**), 60% MeOH (**D**), 80% MeOH (**E**), 100% MeOH (**F**), and 100% EtOH (**G**).

**Figure 5:**
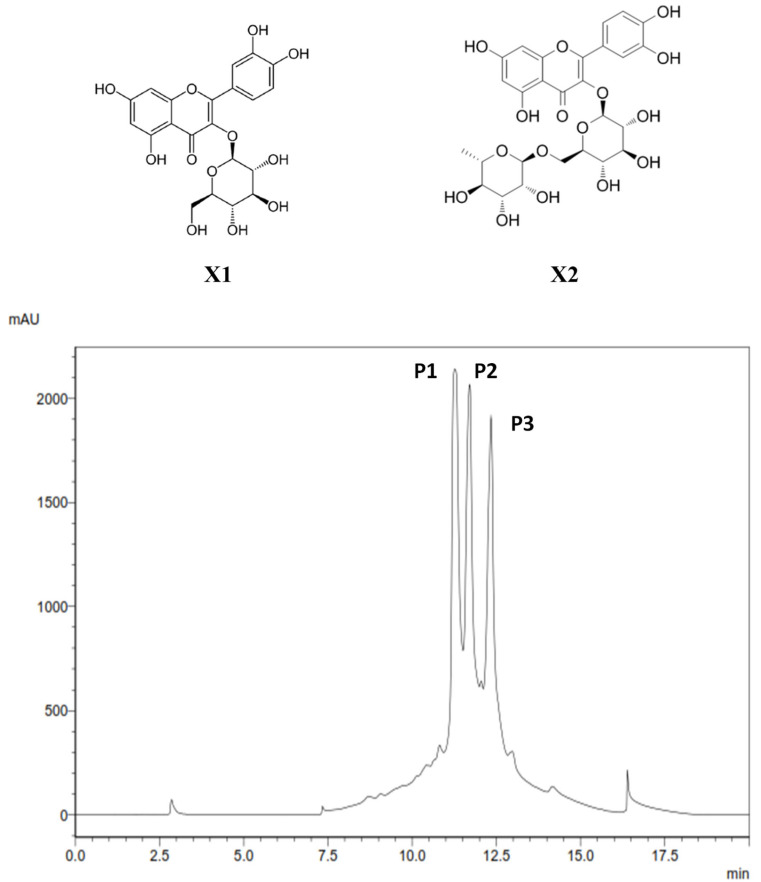
HPLC chromatogram of MeOH_60_ fraction of *C. soldanella* (detection at 254 nm), and chemical structures of quercetin-3-*O*-glycoside (isoquercitrin, X1) and quercetin-3-*O*-rutinoside (rutin, X2).

**Figure 6:**
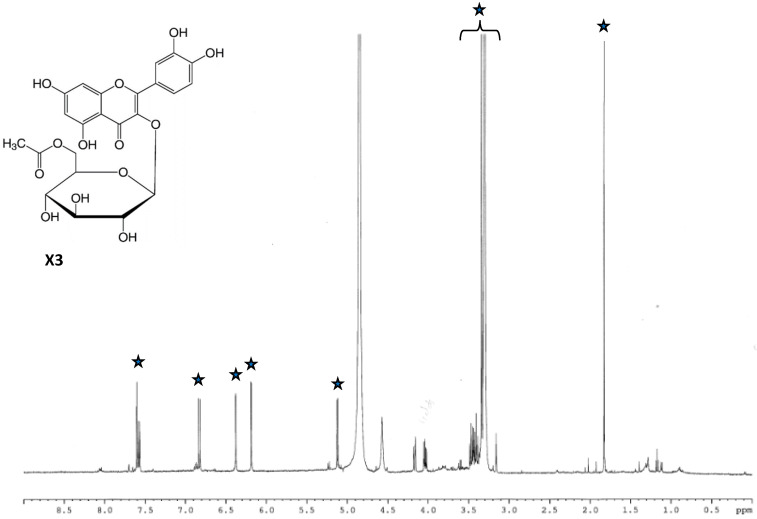
^1^H NMR spectrum of SF3 sub-fraction of MeOH_60_ fraction of *C. soldanella* (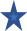) signals and chemical structure of quercetin-3-*O*-β-glucose-6″-acetate, X3).

After fractioning the raw extract, sucrose and glucose were recovered in the water fraction (effluent), as well as quinic acid (Figure 4A). MeOH_20_ fraction mainly showed signals of free and bound forms of quinate (multiplet at 2.1–2.2 ppm), as well as doublets at 6.3 and 7.6 ppm, indicating the presence of chlorogenic acid (Figure 4B). A multitude of small signals were found in the spectrum of the MeOH_40_ fraction, particularly in the aromatic region, but no major compound could be assigned easily from this spectrum (Figure 4C). Conversely, the complex ^1^H-NMR spectrum of the MeOH_60_ fraction exhibited several characteristic signals between 6.1 and 7.6 ppm, which mark the presence of aromatic compounds (Figure 4D), some of which are likely bound to sugars, as evidenced by the signals in the carbohydrate zone (3.2–3.8 ppm). The MeOH_80_ and MeOH_100_ fractions showed relatively similar spectra, with several major signals in the aliphatic region (0.9–1.6 ppm) and a characteristic multiplet of anomeric protons (5.3 ppm) (Figure 4E,F). The last fraction, eluted with 100% ethanol, exhibited signals of aliphatic protons (0.8–1.5 ppm) and a multiplet at 5.3 ppm, as well as small signals in the aromatic region (6.2–7.6 ppm) (Figure 4G). Further purification of the MeOH_60_ fraction by HPLC provided three major peaks upon detection at 254 nm, named P1, P2, and P3 (Figure 4). NMR analysis of P1, P2, and P3 showed marked signals in the aromatic region (5–8 ppm) and some other ones assigned to protons on hydroxylated carbons (3–4.5 ppm). Therefore, these compounds were further investigated with 2D-NMR:

- Upon ^1^H-^1^H COSY experiment, it was possible to identify a first spin system for P1, consisting of six signals at 3.38, 3.46, 3.6, 3.79, 4.5, and 5.1 ppm (Supplementary Data Figure S1). ^1^H-^13^C HMQC and *J*-MOD experiments revealed that protons and carbons of this major constituent were correlated and allowed the identification of a phenolic compound bound to a deoxyose residue. The latter provided both rhamnose and glucose after acid hydrolysis. Finally, using the HMBC experiment, the major compound of the first sub-fraction was identified as quercetin-3-*O*-rutinoside, also known as rutoside and sophorin (Figure 5, X1).
- A similar thorough investigation was conducted on the second sub-fraction (P2) collected by HPLC. Briefly, ^1^H-NMR showed the same signals in the aromatic region as those for X1, suggesting the presence of quercetin. Moreover, 2D-NMR experiments confirmed the nature of the flavonoid as quercetin (Supplementary Data Figure S2), and acid hydrolysis produced glucose residue. Therefore, the major constituent of the second sub-fraction was identified to be quercetin-3-*O*-glucoside (Figure 5, X2).

- ^1^H-NMR spectrum of the third sub-fraction (P3) showed several marked signals in the aromatic region, corresponding to quercetin (Figure 6). Moreover, previous 2D-NMR sequences confirmed the presence of a quercetin derivative (Supplementary data Figures S3 and S4), and acid hydrolysis provided a glucose residue and an acetate group. All together, the information collected for P3 allowed the identification of quercetin-3-*O*-glucose-6″-acetate as the major constituent of the third sub-fraction (Figure 6, X3).

### 2.4. Effect of Identified Molecules on IPEC-J2 Cells upon Mycotoxin Exposure

**Figure 7:**
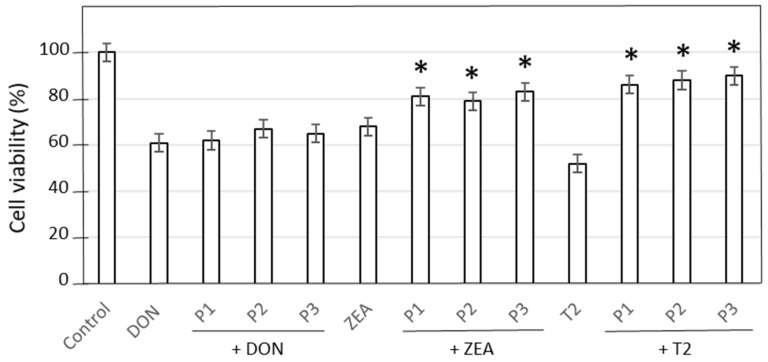
Effect of P1-, P2-, and P3-enriched sub-fractions of *C. soldanella* leaf extract on IPEC-J2 cell viability upon exposure to DON, T2, or ZEA mycotoxins. Mean ± SD of 3 replicates is represented; asterisks indicate a significant difference with the corresponding mycotoxin-treated cells at *p* < 0.05.

The three enriched sub-fractions obtained from the MeOH_60_ fraction of *C. soldanella* leaf extract were tested in the context of mycotoxin contamination (Figure 7). As mentioned above, for the fractions, P1, P2, and P3, sub-fractions had no effect on DON-induced cytotoxicity. Conversely, they significantly mitigated the viability loss caused by T2 or ZEA toxins.

## 3. Discussion

The management of mycotoxins has a significant financial impact on feed producers as well as middlemen like elevators, grain purchasers, and food processors. The toxicological effects of *Fusarium* toxins in farm animals have been widely documented [27,28]. After consuming wheat tainted with deoxynivalenol, cows—the animal type least vulnerable to DON—developed feed refusal syndrome, whereas leukopenia, vomiting, diarrhea, bleeding, shock, and death are among the symptoms observed in pigs [29]. Alimentary Toxic Aleukia (ATA) is the most common manifestation of T-2 toxin’s toxic consequences. Compared to monogastric animals, ruminants are known to be more resistant to T-2 toxin. Ovarian atrophy and vulvar enlargement are linked to the harmful consequences of zearalenone in cows. Because ruminal microbiota can convert ZEA into its hydroxyl-metabolites, α-zearalenol and β-zearalenol, ruminants are less vulnerable to this toxin than pigs.

Many biologically active substances, especially secondary metabolites, have been discovered over decades of searching for natural products in plants. These compounds have been shown to give plants a variety of biological activities. In that context, we have previously reported that halophytic plants may exert cytoprotective and antioxidant effects on animal cell lines upon mycotoxin exposure [22]. Here, the action of sea bindweed (*Calystegia soldanella* L.) was further investigated to elucidate the mechanisms of cell protection and identify its potent bioactive compounds.

Sea bindweed leaf extract exhibited high antioxidant capacities (IC_50_ < 80 µg·mL^−1^ for DPPH radical and TAC of about 80 mg AAE·g^−1^ DW), confirming that halophytes constitutively have a strong antioxidant equipment, which, in turn, enables them to cope with harmful coastal environments [21,30]. Moreover, *C. soldanella* crude extract showed a significant protective action on MDBK and IPEC-J2 cells against ZEA or T2 toxin contamination (restoring about 75% of cell viability at 10 µg·mL^−1^). However, the extract failed to protect animal cells against DON intoxication, suggesting that cell protection upon mycotoxin exposure would require additional mechanisms along with antioxidant action. The three mycotoxins, particularly T2, induced inflammation processes in MDBK and IPEC-J2, but *C. soldanella* crude extract attenuated IL-8 production to control levels. Such anti-inflammatory action from a halophytic plant has already been mentioned [21]. However, it is reported here for the first time in Convolvulaceae.

To understand the cellular mechanism behind this beneficial effect, attention was then paid to the IPEC-J2 model since mycotoxin ingestion by animals (or humans) primarily affects digestive cells. *C. soldanella* crude extract reduced the formation of reactive oxygen species (ROS) in intestinal cells exposed to DON, confirming the antioxidant activity mentioned above. Moreover, it restored calcium flux and epithelial barrier in these cells, indicating a protective effect on cell membranes. Such a protective effect of a halophytic plant extract on intracellular calcium fluxes and membrane stabilization has never been reported hitherto. The normalization of calcium fluxes reflects the preservation of intracellular signaling mechanisms and ionic homeostasis, while the reduction in ROS formation mitigates the oxidative stress induced by mycotoxin, which is commonly associated with cellular damage, inflammation, and apoptosis [31]. Interestingly, though *C. soldanella* reduces ROS formation in cells exposed to T2-toxin, it had no effect on calcium fluxes. This observation suggests that the cytoprotective effect of plant extract is likely mediated through calcium-independent mechanisms. It could be that the extract acts as a direct antioxidant by scavenging ROS or chelating pro-oxidant metal ions, thereby reducing oxidative stress, without altering calcium signaling. It might also exert mitochondrial protection by stabilizing mitochondrial membranes and improving electron transport chain efficiency, which limits electron leakage and ROS production. *C. soldanella*, likely through some of its bioactive compounds, mitigates the toxic impact of mycotoxins on intestinal cells by counteracting their mechanisms of action and limiting damage to cellular membranes, mitochondria, or calcium channels. Moreover, restoration of calcium fluxes implies preservation of intestinal epithelial integrity and barrier function, as calcium plays a critical role in tight junction regulation and epithelial permeability [32]. Overall, the plant constituents may act as direct antioxidants and/or as modulators of cellular signaling pathways involved in oxidative stress and calcium homeostasis, highlighting their potential as protective agents for intestinal health.

Fractionation of the crude extract to identify the main compounds of sea bindweed and their biological activities yielded six fractions. Among them, the fraction eluted with MeOH_60_ showed the most significant antioxidant and cytoprotective effects. 1D and 2D NMR studies identified three predominant compounds in this fraction. These compounds were all found to be glycosylated quercetins: quercetin-3-*O*-glucose, quercetin-3-*O*-rutinoside, and quercetin-3-*O*-glucose-6″-acetate. Quercetin-3-*O*-glucose, also known as isoquercitrin, is a flavonol-type flavonoid that is widely represented in plants. In particular, in halophytic plants, it was found in species like *Avicennia marina, Crithmum maritimum, Salicornia ramosissima,* or *Vachellia raddiana* [33,34,35,36,37]. Its presence has been reported in the Convolvulaceae family too, including in *C. soldanella* [38]. Quercetin-3-*O*-rutinoside, also known as rutin or sophorin, is quite commonly encountered in plants, with the most rutin-rich plants being *Capparis spinosa*, *Olea europeae*, *Fagopyrum esculentum*, *Asparagus officinalis*, and *Rubus* sp. [24]. In halophytic plants, rutin has been reported in *Anabasis ehrenbergii*, *Crithmum maritimum*, or *Zygophyllum album* [37,39,40]. In Convolvulaceae, it has been previously described in *Convolvulus arvensis* [41], and Murai et al. [38] also mentioned it in sea bindweed. This flavonol has been shown to possess antiaggregant and choleretic properties, and it may be used to treat capillary weakness or Parkinson’s disease [24]. Finally, quercetin-3-*O*-glucose-6″-acetate has only been reported in the glycophytes *Hymenoxys hoopesii* (Asteraceae), *Morus alba* (Moraceae), and *Peucedanum ostruthium* (Apiaceae) [42,43]. Here, the presence of this flavonol is reported for the first time in a halophytic species and in the Convolvulaceae family.

The powerful antioxidant and anti-inflammatory properties of quercetin and its glycosylated derivatives have been well documented [44,45,46,47]. Moreover, the flavonol and its glycosides have been shown to inhibit acetylcholinesterase (AChE) activity and enhance choline acetyltransferase (ChAT) activity, leading to cholinergic neurotransmission and thereby improving neurodegenerative disease [48,49]. However, information regarding the potent protective activity of quercetin derivatives against mycotoxins is scarce and only concerns the aglycone [50]. Indeed, a quercetin-supplemented diet has been shown to be beneficial in protecting against exposure to mycotoxins [51,52,53]. However, to our knowledge, the potential antimycotoxin effects of glycosylated quercetins have never been reported. Since glycosylated quercetines or similar flavonols are abundant in halophytes, these plants appear as promising new natural sources to prevent mycotoxin hazards. The confirmation of such benefits for domestic livestock or humans still requires testing these purified compounds in vivo.

## 4. Conclusions

Overall, sea bindweed leaves and some of their enriched fractions were found to have antioxidant and anti-inflammatory activities, along with cytoprotective effects on animal cell lines upon mycotoxin exposure. This work shows that sea bindweed could find dietary uses, as an interesting source for feed ingredients, to prevent potential intoxication with fungal toxins. Further work should be conducted to test the potential curative effect of plant extract or derived compounds on food intoxication, and to validate in vivo the results observed here. Alternatively, seasonal and organ variability of biochemical contents and bioactivities in *C. soldanella* plants sampled from their natural habitat or grown in an open field should also be investigated.

## 5. Materials and Methods

### 5.1. Chemicals, Culture Media, and Supplements

The intestinal porcine enterocyte (IPEC-J2, ACC 701) and Madin–Darby bovine kidney (MDBK, ACC 174) cell lines were provided by DSMZ (Braunschweig, Germany). The cell culture media (DMEM, EMEM), horse fetal serum (HSF), Folin–Ciocalteau phenol reagent, DPPH reagent, and all standards and solvents needed for chemical analyses were purchased from Sigma Aldrich (St. Louis, MO, USA), as well as deoxynivalenol (DON), T2 toxin, and zearalenone (ZEA) mycotoxins.

### 5.2. Cell Culture

MDBK and IPEC-J2 cell lines were selected as complementary models for studying mycotoxin toxicity. MDBK cells, derived from bovine kidney tissue, are used to assess systemic cytotoxicity and cellular sensitivity to mycotoxins after absorption, as the kidney is an important target organ for their distribution. IPEC-J2 cells, derived from non-tumorous porcine intestinal epithelium, represent a relevant model of the intestinal barrier, the primary site of exposure to mycotoxins after ingestion. The combined use of these two cell lines thus makes it possible to understand both the initial and systemic effects of mycotoxins, as well as the potential efficacy of protective compounds in physiologically complementary contexts. Here, IPEC-J2 cells were cultivated in Dulbecco’s Modified Eagle Medium (DMEM) with 1% HEPES, 10% FBS, and 1% penicillin/streptomycin added as supplements. MDBK were cultivated in Eagle’s Minimum Essential Medium (EMEM) that contained 4.5 g/L glucose for cell growth, 10% horse fetal serum (HSF), and 1% penicillin/streptomycin. Both MDBK and IPEC-J2 cells were cultivated at 37 °C under 5% CO_2_.

### 5.3. Plant Sampling

Aerial parts of sea bindweed were collected in July 2022 on sand dunes of Finistère (France) at Le Conquet (48°22′14.0″ N 4°45′43.8″ W). One sample was deposited at the herbarium of the University of Brest (n°Cs27). The collected samples were rapidly rinsed to remove sand, and the leaves were frozen and subsequently freeze-dried for 48 h at −56 °C (YR05186 freeze-dryer, Kalstein, Paris, France). The dry sample was then ground to a fine powder in a Mettler AE 200 blender (Mettler, Viroflay, France) before extraction and analysis.

### 5.4. Extraction of Metabolites from Calystegia soldanella Leaves

About 500 mg of leaf powder was mixed with 5 mL of water/ethanol (1:2) under magnetic stirring at 4 °C for 20 min. The mixture was centrifuged for 15 min at 4 °C and 4000× *g*, and the resultant pellet was then extracted twice using the same procedure. The supernatants were collected, pooled, and filtered over glass wool. The obtained extract was concentrated by rotary evaporation at 40 °C and resuspended in either DMSO (for cell treatments) or 50% ethanol (for purification and biochemical analyses).

### 5.5. Evaluation of Calystegia soldanella Extract Effects on Cell Lines

#### 5.5.1. Cell Treatments

In 96-well plates, MDBK and IPEC-J2 cells were seeded at 5 × 10^5^ cells/mL. Following one day of culture, plant extracts were added (0.1–10 μg·mL^−1^). One day later, cells were incubated with cytotoxic concentrations of mycotoxins (DON at 2 × 10^−5^ M, ZEA at 5 × 10^−9^ M, or T2 at 10^−4^ M) for 48 h before viability analysis. These concentrations were established based on preliminary dose–response experiments [54].

#### 5.5.2. Evaluation of Cell Viability

The cytotoxic impact of sea bindweed polar extract on MDBK and IPEC-J2 cells was investigated according to Hymery et al. [54]. Cytotoxicity was evaluated using the Cell Titer 96AQueous One cell proliferation assay (Promega, Madison, WI, USA), which assesses the conversion of 3-(4,5-dimethylthiazol-2-yl)-5-(3-carboxymethoxyphenyl)-2-(4-sulfophenyl)-2H-tetrazolium (MTS) to formazan by mitochondrial dehydrogenases in viable cells. Cells incubated for 24 h with samples (control or plant extract) and then for 48 h with mycotoxin were washed with PBS, resuspended in 100 μL of the same buffer, and seeded in 96-well plates. They were then incubated at 37 °C, with 5% CO_2_ atmosphere and 100% humidity for 3 h. Following the addition of 20 μL of CellTiter 96AQueous One solution to each well, the cells were incubated for an additional three hours at 37 °C with 5% CO_2_. The absorbance was measured at 490 nm, and cytotoxicity was expressed as the percentage of cell viability compared with the control (cells treated with 1% DMSO).

#### 5.5.3. Measurements of Cytokine Production

The production of interleukine-8 (IL-8) was tracked to assess the mycotoxin and plant extract’s potential inflammatory impact on MDBK and IPEC-J2 cells. Cells were cultured with *C. soldanella* extract for 24 h, then with mycotoxins for an additional 24 h. Thereafter, ELISA kits (Promocell, Heidelberg, Germany) were used to measure the levels of IL-8 in the supernatants of cell cultures. Results were expressed as the ratio of IL-8 produced in mycotoxin- or plant extract-treated cells over control ones.

#### 5.5.4. Measurements of Reactive Oxygen Species in Treated Cells

Cells were cultured in 12-well plates at a concentration of 3 × 10^5^ cells/mL. After 5 days of exposure to mycotoxins only or with sea bindweed extract, the amount of reactive oxygen species (ROS) in the cells was measured with the CellROX Green^®^ reagent. Under normal conditions, this reagent is non-fluorescent, but upon oxidative stress, CellROX Green^®^ emits a strong fluorescent signal, allowing the measurement of ROS levels in live cells. Each well was collected and centrifuged, and the supernatant was discarded. The cells were then resuspended in PBS. A negative control consisted of cells incubated with 2 mM N-acetylcysteine (NAC) for 1 h at 37 °C under 5% CO_2_. Thereafter, each sample was incubated with tert-butyl hydroperoxide (TBHP) at a final concentration of 200 µM for 1 h at 37 °C under 5% CO_2_. CellROX Green^®^ was then added at a final concentration of 500 µM, and the mixture was left for 1 h at 37 °C under 5% CO_2_. During the last 15 min of staining with CellROX, the SYTOX^®^ Red Dead Cell reagent was added to stain necrotic cells. Cells were subsequently analyzed by flow cytometry (BD ACCURI C6, BD Medical, Franklin Lakes, NJ, USA) using the following excitation wavelengths: 488 nm for CellROX^®^ Green and 639 nm for SYTOX^®^ Red Dead Cell.

#### 5.5.5. Measurements of Intracellular Calcium

The fluorescent probe Fura2-QBT was utilized to quantify variations in intracellular calcium concentration. A calcium chelator was added to this cell-permeable probe, enabling it to bind calcium. The probe emitted at 510 nm after being stimulated at 340 and 380 nm. Because Fura2 binds to Ca^2+^, an increase in cytoplasmic calcium concentration causes an increase in fluorescence intensity at 340 nm. On the other hand, as the concentration of Fura2’s unbound form decreased, the fluorescence intensity at 380 nm decreased. The intracellular Ca^2+^ level was then quantitatively measured by calculating the ratio of the two fluorescence intensities that resulted from excitation at 340 nm and 380 nm.

Calcium flux measurement was performed using the FlexStation. Cells were seeded in 96-well plates at a density of 2 × 10^5^ cells/mL, and calcium flux was assessed after 5 days of exposure to mycotoxins only or with *C. soldanella* extract. The plates were placed individually into the FlexStation, and a first injection of 1 µM thapsigargin was performed at 100 s, followed by a second injection of a 10 mM Ca^2+^ solution (25 µL) at 500 s. Thapsigargin acts on the SERCA pumps, causing the release of calcium stores from the endoplasmic reticulum into the cytoplasm. Since the buffer in the wells contained no Ca^2+^, any increase in the signal indicated calcium release from the cells. After the second injection, calcium flux was measured via the store-operated calcium channels (SOCs). Indeed, with calcium store depletion, SOCs were activated to allow Ca^2+^ entry into the cell. Intracellular Ca^2+^ was quantified by calculating the ratio of fluorescence intensities at 340 nm and 380 nm excitation. The amplitudes of the resulting curves were then compared.

### 5.6. Measurement of Antioxidant Activities in Calystegia soldanella Extract

#### 5.6.1. Total Antioxidant Capacity (TAC)

According to Prieto et al. [55], the assay of a green phosphate/Mo5+ complex was used to assess the total antioxidant capacity of sea bindweed ethanolic extract. One mL of reagent solution (0.3 N sulfuric acid, 28 mM sodium phosphate, and 4 mM ammonium molybdate) was added to an aliquot (0.1 mL) of diluted extract or to methanol as the blank. The tubes were incubated in a boiling water bath for 90 min, then cooled to room temperature, and their absorbance was measured at 695 nm against a blank in a UV-Visible spectrophotometer (Anthelie Advanced 2, Secoman). Antioxidant capacity was assayed in technical triplicates and expressed as mg ascorbic acid equivalent per gram of dry weight (mg AAE·g^−1^ DW). All samples were analyzed in triplicate.

#### 5.6.2. DPPH Scavenging Activity

The scavenging activity of *C. soldanella* extract on the 1,1-diphenyl-2-picrylhydrazyl (DPPH) free radical was determined according to Marwah et al. [56]. One hundred microliters of 100 μM DPPH violet solution in ethanol and 100 μL of plant extract at varying concentrations (or water for the control) were mixed. After 15 min of dark incubation, the absorbance of the reaction mixture was measured at 517 nm using a microtiter reader (Multiskan EAR 400, Labsystems, Mumbai, India). The assay was carried out in triplicate, and butylated hydroxytoluene (BHT) was used as a positive control. The inhibition percentage (%IP) of the DPPH radical was determined by measuring the drop in absorbance upon the addition of test samples, with the following equation:

(1) $$\%\mathrm{IP}=\left[(A_{c}-A_{s})/A_{c}\right]\times 100$$

where A_c_ and A_s_ are the absorbances of the control and the test sample, respectively. The antiradical activity, represented by the extract concentration that results in a 50% inhibition (IC_50_), was calculated for each sample using a linear regression analysis using a plot of concentration against %IP (GraphPad Prism v. 5.0 program).

### 5.7. Fractionation of Calystegia soldanella Extract

Solid–liquid partition chromatography was used to fractionate the raw extract of *C. soldanella* on C18-bound silica gel. Increasing methanol concentrations (successively 0, 20, 40, 60, 80, and 100%) were used to elute polar molecules, followed by 100% ethanol. The collected fractions were then concentrated by rotary evaporation at 40 °C and reconstituted in the appropriate solvent.

### 5.8. Solute Purification

Further purification from a single fraction was carried out by HPLC using a Shimadzu UFLC XR device fitted with a PDA detector (SPD-M20A, Shimadzu, Kyoto, Japan). The mobile phase was a combination of 100% acetonitrile (A) and ultrapure water (B), and solutes were separated using a Spherisorb ODS2 column (5 μm, 250 × 4.6 mm, Waters) with the following linear gradient: t = 0 min 100% B; t = 10 min 100% A; t = 12 min 100% A; and t = 15 min 100% B. Each compound detected at 254 nm was collected. When needed, some of them were submitted to acid hydrolysis treatment (1 N HCl, 110 °C for 1 h) before structural elucidation.

### 5.9. NMR Analyses

Aliquot of *C. soldanella* crude extract and each fraction was concentrated by rotary evaporation at 35 °C, and the dry residue was solubilized in deuterated-water (D_2_O) or methanol (MeOD) for compound characterization with NMR analyses. ^1^H NMR spectra were obtained on a Brüker Avance DRX-400 spectrometer (400 MHz), fitted with a 5 mm TBI probe (^1^H, X, ^31^P) with z gradient (Brüker, Rheinstetten, Germany). A typical 1D ^1^H NMR spectrum consisted of 32 scans. The determination of major solutes present in sea bindweed extract or fractions was made on NMR spectra in comparison with external standards. All ^13^C (J-mod) and 2D Homo- and heteronuclear NMR analyses (COSY, HMBC, and HMQC experiments) were performed on a Brüker Avance III HD500 spectrometer equipped with a 5 mm TCI cryoprobe (^1^H, ^13^C, ^15^N) with z gradient. Data analysis was performed using TopSpin^®^ software, 4.0 (Brüker).

### 5.10. Statistical Analyses

Every extraction and assay was replicated three times. The results were presented as mean ± standard deviation (SD), and the means were compared by one-way analysis of variance (ANOVA) followed by Duncan’s multiple range tests using the ‘‘Statistica v. 5.1’’ software (Statsoft, 2008). Individual mean differences were considered significant at *p* < 0.05.

## Figures and Tables

**Table 1:** Antioxidant activities of *C. soldanella* leaf extract and its methanol and ethanol fractions using total antioxidant capacity (TAC) and DPPH-quenching bioassays. Data are mean ± SD of 3 replicates. In each column, different letters indicate significant differences between the samples at *p* < 0.05.

	TAC (mg AAE·g^−1^ DW )	DPPH (IC_50_, µg·mL^−1^)
Raw extract	89.1 ± 1.4 c	77.5 ± 6.0 b
MeOH_20_	175.5 ± 13.7 a	20.9 ± 1.9 d
MeOH_40_	179.3 ± 13.8 a	18.1 ± 0.5 d
MeOH_60_	200.1 ± 9.7 a	13.8 ± 0.5 e
MeOH_80_	133.9 ± 17.7 b	45.8 ± 1.5 c
MeOH_100_	17.2 ± 6.9 d	ND (>1000) a
Ethanol_100_	85.7 ± 19.9 c	969.1 ± 25.0 a

**Table 2:** Effect of *C. soldanella* leaf extract (1 µg·mL^−1^) on IPEC-J2 cell viability percentage upon DON, ZEA, or T2-toxin exposition (10 µM each). Data are mean ± SD of 3 replicates; in each line, a letter indicates a significant difference with the mycotoxin-treated control cells at *p* < 0.05 (a) and *p* < 0.01 (b).

Cell Line	Mycotoxins	Control	+Raw Extract
MDBK	DON	60.6 ± 1.1	61.1 ± 1.3
	ZEA	61.9 ± 1.2	75.3 ± 0.7 a
	T2	51.5 ± 1.4	74.0 ± 1.2 b
IPEC-J2	DON	60.5 ± 1.2	61.1 ± 1.3
	ZEA	62.0 ± 1.2	75.3 ± 0.7 a
	T2	52.1 ± 1.4	76.4 ± 1.9 b

**Table 3:** Effect of *C. soldanella* extract (1 µg·mL^−1^) on interleukine-8 production in MDBK and IPEC-J2 cells exposed to DON, ZEA, and T2 toxins. Data, expressed as a ratio to untreated cells, are mean ± SD of 3 replicates. In each line, a letter indicates a significant difference with the mycotoxin-treated control cells at *p* < 0.05.

Cell Line	Mycotoxins	Control	+Raw Extract
MDBK	DON	3.61 ± 0.21	1.08 ± 0.05 a
	ZEA	4.12 ± 0.23	1.09 ± 0.06 a
	T2	7.33 ± 0.41	1.41 ± 0.12 a
IPEC-J2	DON	3.74 ± 0.20	1.04 ± 0.03 a
	ZEA	3.32 ± 0.18	1.07 ± 0.07 a
	T2	5.21 ± 0.39	1.20 ± 0.16 a

**Table 4:** Effect of *C. soldanella* extract (1 µg·mL^−1^) on store-operated Ca^2+^ channel function in IPEC-J2 cells exposed to DON, ZEA, and T2 toxins. Data, expressed as a percentage of the SOC function in untreated cells, are mean ± SD of 3 replicates. In each line, a letter indicates a significant difference with the mycotoxin-treated control cells at *p* < 0.05 (a) and *p* < 0.01 (b).

Mycotoxins	Control	+Raw Extract
DON	65.6 ± 1.1	95.1 ± 2.3 b
ZEA	33.7 ± 2.3	45.8 ± 3.1 a
T2	51.9 ± 1.2	53.3 ± 4.7

**Table 5:** Effect of *C. soldanella* crude extract (1 µg·mL^−1^) on trans-epithelial electrical resistance (TEER) in IPEC-J2 cells exposed to DON, ZEA, and T2 toxins. Data, expressed as % of membrane resistance (100% for intact cells), are mean ± SD of 3 replicates. In each line, a letter indicates a significant difference with the mycotoxin-treated cells (control) at *p* < 0.05 (a) or *p* < 0.01 (b).

Mycotoxins	Control	+Plant Extract
DON	79 ± 4	89 ± 5 a
ZEA	75 ± 6	95 ± 5 a
T2	66 ± 4	92 ± 6 b

## Data Availability

The original contributions presented in this study are included in the article and/or Supplementary Materials. Further inquiries can be directed to the corresponding author.

## Supplementary Materials

The following supporting information can be downloaded at: https://www.mdpi.com/article/10.3390/toxins18030127/s1. Figure S1: ^1^H-^1^H COSY correlation of SF1 sub-fraction of MeOH_60_ fraction of *C. soldanella*; Figure S2: ^1^H-^1^H COSY correlation of SF2 sub-fraction of MeOH_60_ fraction of *C. soldanella* (first spin system in green, second one in blue); Figure S3: ^1^H-^1^H COSY correlation of SF3 sub-fraction of MeOH_60_ fraction of *C. soldanella* (first spin system in green, second one in blue); Figure S4: 2D HMQC ^1^H-^13^C NMR spectrum of SF3 sub-fraction of MeOH_60_ fraction of *C. soldanella*.

## Abbreviations

AAE, ascorbic acid equivalent; DMSO, dimethyl sulfoxide; DON, deoxynivalenol; DPPH, 2,2-diphenyl-1-picrylhydrazyl; DMEM, Dulbecco’s Minimum Essential media; EMEM, Eagle’s Minimum Essential Medium; EtOH, ethanol; HSF, horse fœtal serum; IC_50_, inhibitory concentration of 50%; IPEC-J2, intestinal porcine cell line; MDBK, bovine kidney cell line; MeOH, methanol; MTS, 3-(4,5-dimethylthiazol-2-yl)-5-(3-carboxymethoxyphenyl)-2-(4-sulfophenyl)-2H-tetrazolium; NO, nitric oxide; ZEA, zearalenone.
