# Supplementary material for: Sea Bindweed Prevents Mycotoxin Intoxication Through Antioxidant, Anti-Inflammatory and Cytoprotective Activities
Source: Toxins (Basel). 2026 Mar 2;18(3):127. doi: 10.3390/toxins18030127 (PMC13029956; doi:10.3390/toxins18030127)
Supplement: Supplementary file 1 [file toxins-18-00127-s001.zip › toxins-4127487-supplementary.pdf]

Supplementary Materials: Sea Bindweed Prevents Mycotoxin Intoxication Through Antioxidant, Anti-Inflammatory and Cytoprotective Activities

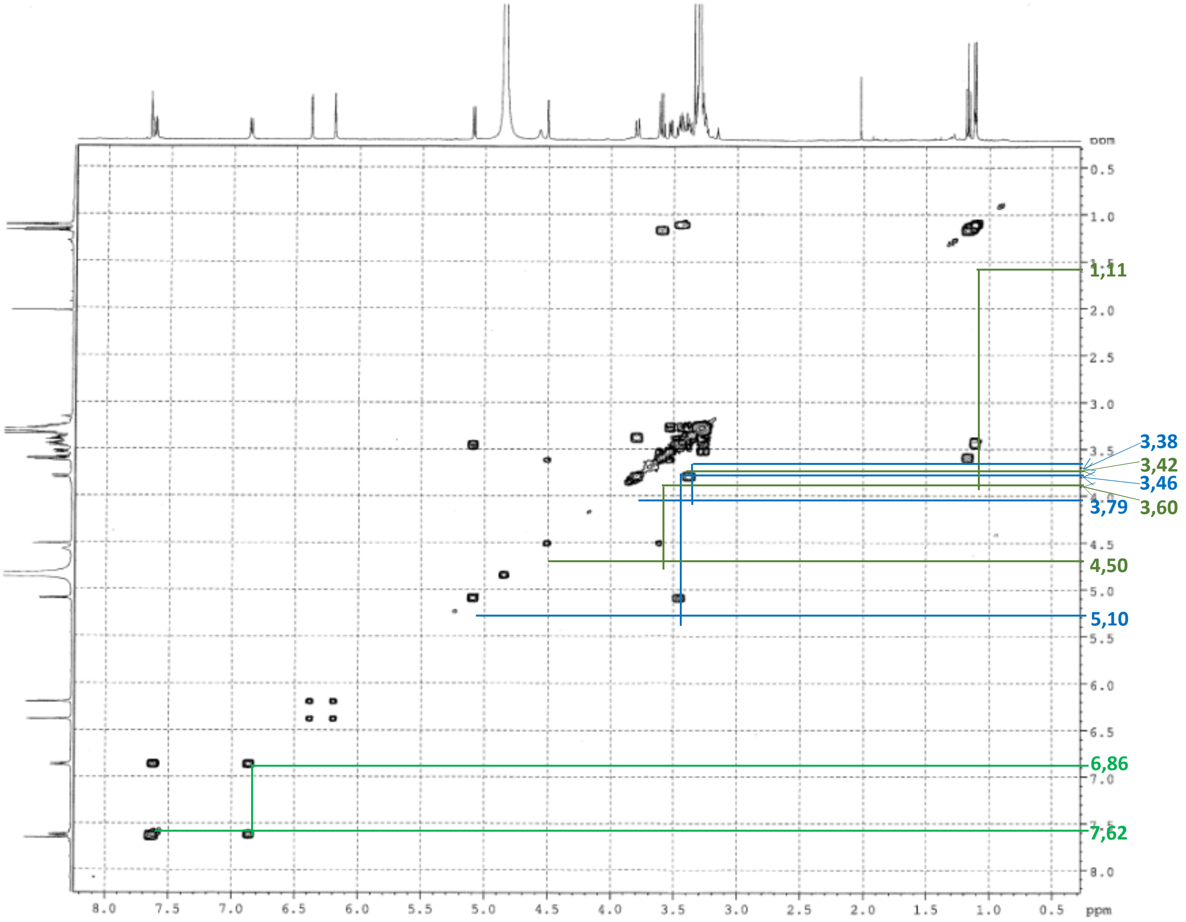

Figure S1: ^{1}H-^{1}H COSY correlation of SF1 sub-fraction of $MeOH_{60}$ fraction of *C. soldanella* (first spin system in green, second one in blue).

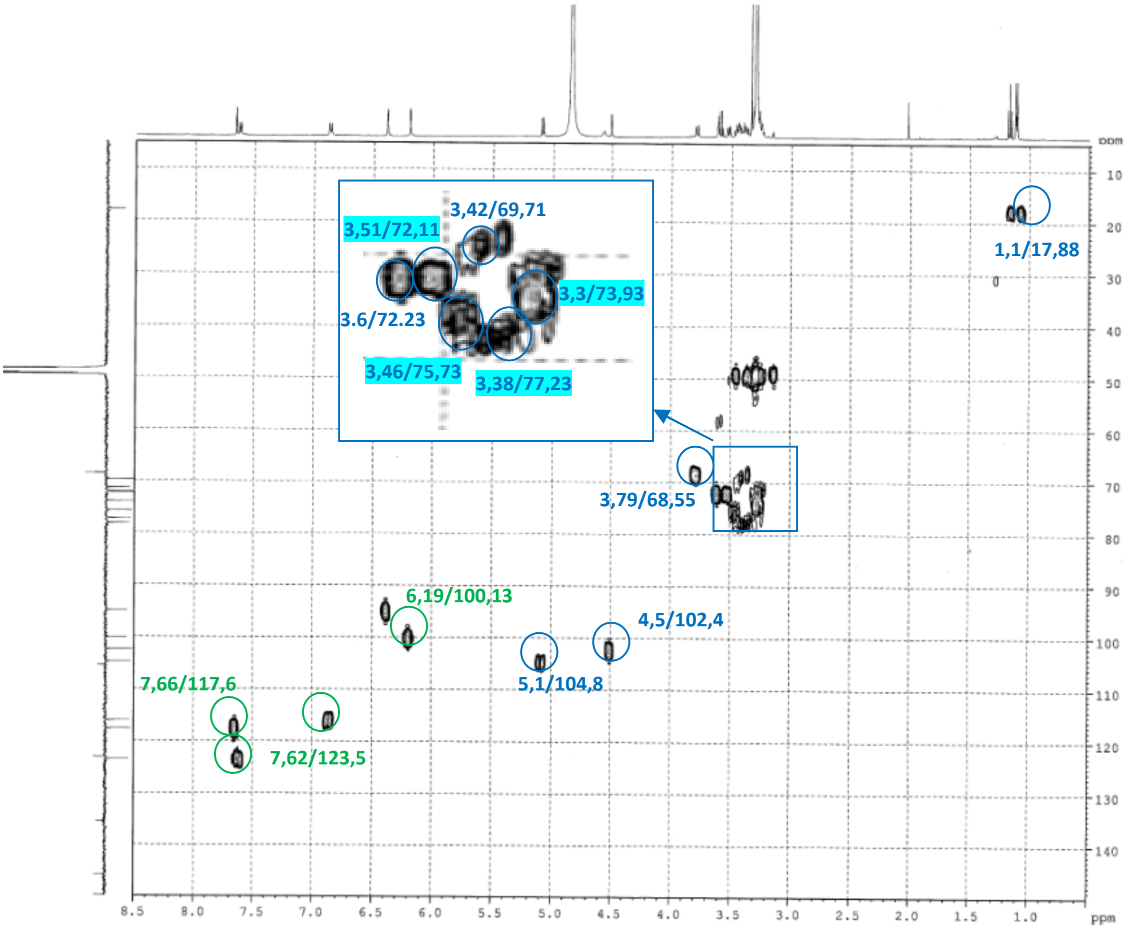

Figure S2 : ^{1}H-^{1}H COSY correlation of SF2 sub-fraction of $MeOH_{60}$ fraction of *C. soldanella* (first spin system in green, second one in blue).

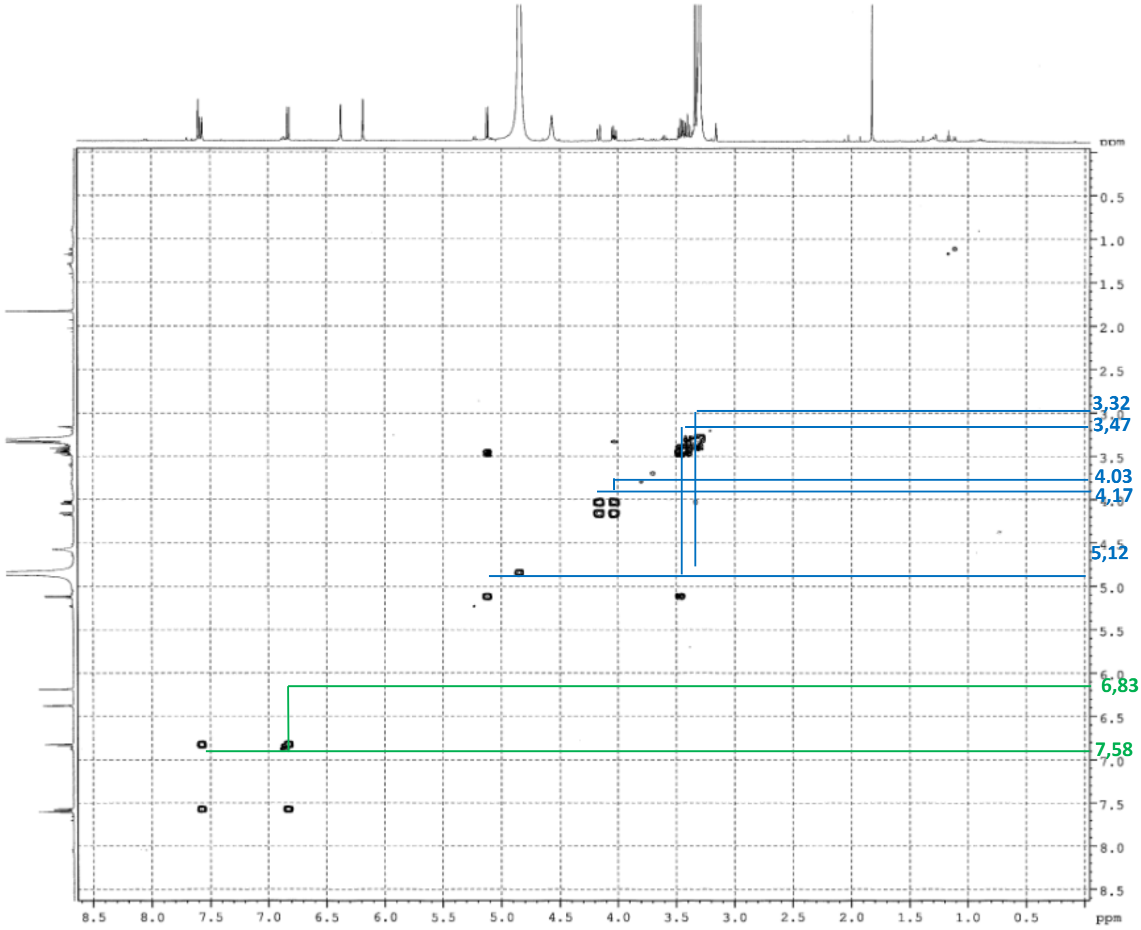

Figure S3 : ^{1}H-^{1}H COSY correlation of SF3 sub-fraction of $MeOH_{60}$ fraction of *C. soldanella* (first spin system in green, second one in blue).

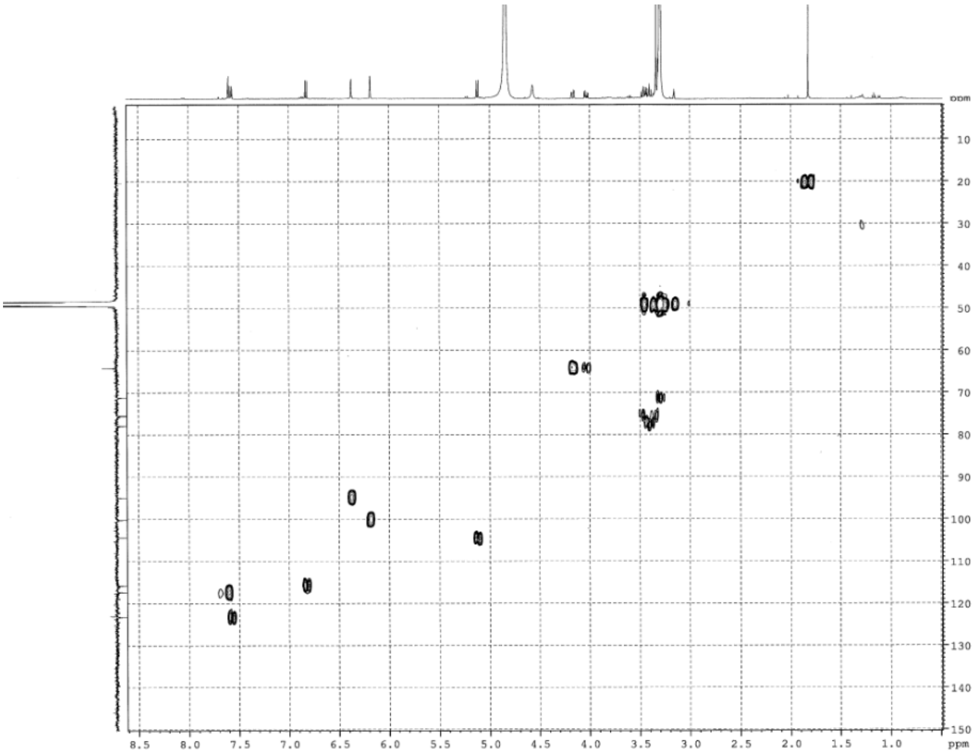

Figure S4 : 2D HMQC ^{1}H-^{13}C NMR spectrum of SF3 sub-fraction of $MeOH_{60}$ fraction of *C. soldanell*a.
